# Endohepatology: Current perspectives and future directions

**DOI:** 10.1097/HC9.0000000000000767

**Published:** 2025-08-15

**Authors:** Ahmad Najdat Bazarbashi, Allison R. Schulman

**Affiliations:** 1 Division of Gastroenterology, King Faisal Specialist Hospital and Research Center, Riyadh, Kingdom of Saudi Arabia; 2 Division of Gastroenterology, Washington University in St. Louis/Barnes-Jewish Hospital, St. Louis, Missouri, USA; 3 Division of Gastroenterology, University of Michigan School of Medicine, Ann Arbor, Michigan, USA

**Keywords:** endoscopic hepatology, endoscopic ultrasound, liver biopsy, liver disease

## Abstract

Endoscopic procedures have been integral to the management of patients with liver disease, primarily focusing on luminal interventions such as variceal band ligation. Since the introduction of endoscopic ultrasound, the breadth of procedures has rapidly expanded, leading to the emergence of a new field known as endoscopic hepatology or “endohepatology.” This domain integrates endoscopic diagnostic and therapeutic techniques, particularly those which are endoscopic ultrasound–based, into hepatology practice. The comprehensive nature of endohepatology has revolutionized patient care by facilitating a streamlined yet comprehensive approach that enhances efficacy, reduces resource utilization and improves patient satisfaction. Patients can now undergo multiple procedures, such as variceal surveillance and endoscopic ultrasound-liver biopsy in a single session. This review explains the current landscape of endohepatology, highlighting established practices, recent technology advancements, and future directions in the field.

## INTRODUCTION

Endoscopic diagnostic and therapeutic interventions have played a pivotal role in the management of patients with liver disease for many decades. While these procedures have predominantly focused on luminal interventions such as variceal band ligation, the endoscopic armamentarium has evolved considerably with advancements in endoscopic ultrasound (EUS). Given the rapid expansion of this field, the term “endoscopic hepatology” or “endohepatology” has been coined.^1^ Endohepatology refers to the overlap or intersection of endoscopic procedures, particularly procedures that utilize echoendoscopy, with the practice of hepatology. These interventions offer alternative routes for the diagnosis and management of patients with liver disease. This field continues to expand rapidly with enhanced understanding of liver disease and advancements in endoscopic technology.

The field of endohepatology has also allowed for the potential of a comprehensive approach in a patient population often subjected to numerous interventions for both the diagnosis and management of liver disease. This approach has streamlined clinical care, decreased resource utilization, and improved patient satisfaction. For example, a patient can undergo variceal surveillance and ligation if present, EUS-guided assessment of the liver stiffness with elastrography, and simultaneous and potentially targeted liver biopsy for histopathological assessment. In this review, we will highlight common practices performed currently in endohepatology and shed light on newer technologies and future perspectives in this ever-evolving field.

## CURRENT PRACTICES IN ENDOHEPATOLOGY

### EUS-guided liver parenchyma biopsy

While noninvasive testing for liver disease has taken tremendous strides over the past several years with improved diagnostic accuracy and decreased need for histological assessment,^2^ liver biopsy remains the gold standard method for assessing liver disease and an important tool in the armamentarium of the hepatologist. EUS-guided liver biopsy (EUS-LBx) has become increasingly popular nationwide, and at some centers has largely replaced the percutaneous approach to become the primary route of hepatic tissue acquisition. EUS-LBx has the potential to significantly streamline patient care when coupled with diagnostic upper endoscopy for evaluation of abdominal pain or for variceal screening or surveillance.

**FIGURE 1:**
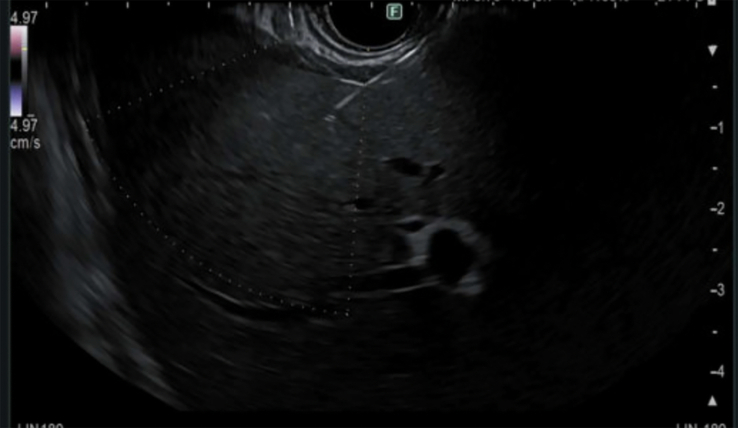
EUS-guided parenchymal liver biopsy using a 22-G core needle. Abbreviation: EUS, endoscopic ultrasound.

The first EUS-LBx was performed in 2006,^3^ and the technique has since evolved and been standardized to provide high-quality cores for histological analysis.^4^ EUS-LBx is typically performed in patients under monitored anesthesia care and in the left lateral decubitus position. When required, an upper endoscopy can be performed prior to the EUS to assess for structural pathology and to look for stigmata of liver disease. After that, a linear echoendoscope is passed into the stomach and small intestine. This allows for clear visualization of both lobes of the liver. Under ultrasound Doppler guidance, intervening vessels such as branches of the hepatic and portal veins can be assessed to ensure they are not in the path of needle access. A core biopsy needle is typically used and advanced under a wet suction technique, with 1–3 needle actuations performed under negative pressure suction (Figure 1). The core acquired is then examined to ensure macroscopic adequacy and should be handled with care to avoid fragmentation. If there is a visual concern that an inadequate core of tissue has been obtained, it is recommended to obtain a second pass from the contralateral lobe of the liver.

The goal of these biopsies is to emulate the best standard of care available in terms of obtaining core tissue for histological assessment. Recommendations and best practices by the American Association for the Study of Liver Diseases (AASLD) highlight that liver biopsies should ideally be at least 20 mm in length and with ≥11 complete portal tracts (CPTs).^5^

Current literature suggests that 19-gauge needle biopsy needles are superior to 22-gauge needles, and if a 19-gauge needle biopsy needle cannot be acquired, a fine needle aspiration (FNA) of the same size is recommended to avoid fragmentation.^6^ Studies have also found that the wet suction technique (priming the needle with saline or heparin) provides better yield than dry suction.^7^ Combining these observations and findings, EUS-LBx can produce cores similar to and comparable to a percutaneous route.

Numerous studies have confirmed the safety and high diagnostic accuracy of EUS-LBx; however, few prospective randomized controlled trials to compare EUS-LBx to percutaneous biopsies have been performed (summarized in Table 1). One recent randomized controlled trial by Bang et al compared percutaneous liver biopsy to EUS-guided liver biopsy and revealed that the percutaneous route yielded significantly more optimal specimens (57.8% vs. 23.8%, *p*=0.028) and was less costly (US$1824 vs. US$3240, *p*<0.001). However, several questions were raised in this study, including an unclear definition of optimal specimen and failure to adopt the saline technique, which is commonly employed to improve biopsy outcomes.

**TABLE 1:** Summary of prospective randomized controlled trials comparing EUS-guided liver biopsy to the percutaneous route

Author name	Year	Design	Sample size	Key outcomes
Bang et al^8^	2021	Single center (United States)	40 patients 20 EUS-LBx 20 Perc-LBx	• Both EUS-LBx and percutaneous liver biopsy achieved 100% diagnostic adequacy. • Percutaneous liver biopsy had a higher proportion of “optimal” biopsies (57.9% vs. 44.4%). • No significant adverse events reported in either group. *** Limitations included the definition of optimal biopsy, an underpowered sample size and the use of a no-suction technique in EUS-LBx.
Ali H. et al^9^	2023	Single center (United States)	80 patients 40 EUS-LBx 40 Perc-LBx	• Diagnostic adequacy was similar between EUS-LBx and percutaneous liver biopsy (92.5% vs. 95%). • Median number of complete portal tracts was higher in Perc-LBx (17 vs. 13). • EUS-LBx was associated with less post-procedure pain (median pain score 2.0 vs. 3.0 and shorter hospital stay (2.0 vs. 4.0 h). • No serious adverse events reported in either group.
Samanta et al^10^	2022	Multicenter (India)	48 patients 24 EUS-LBx 24 Perc-LBx	• EUS-LBx provided significantly longer aggregate specimen lengths and a higher number of complete portal tracts compared to percutaneous liver biopsy. • EUS-LBx patients reported less post-procedure pain. • Both techniques demonstrated similar diagnostic adequacy.
Larino-Noia et al^11^	2023	Single center (Spain)	90 patients 44 EUS-LBx 46 Perc-LBx	• EUS-LBx achieved a higher rate of adequate samples (70.4%) compared to percutaneous liver biopsy. • Total specimen length is longer in the EUS-LBx group (23.5 mm) than in the percutaneous liver biopsy group (17.5 mm). • Sample fragmentation is more frequent in EUS-LBx samples. • No differences in adverse events.

Abbreviations: EUS, endoscopic ultrasound; EUS-LBx, EUS-guided liver biopsy.

A recent meta-analysis published in 2023 included 5 studies (including 2 RCTs) and 748 patients and compared EUS-LBx to percutaneous liver biopsy.^12^ The diagnostic accuracy for EUS-LBx and percutaneous liver biopsy was 88.3% and 98.6%, respectively (OR 1.65, *p*=0.04). The mean total specimen length was statistically higher in the percutaneous liver biopsy group as compared to the EUS-LBx group, with a mean difference of 1.25 cm. The adverse event rate for EUS-LBx was 13% compared to 11.9% in the percutaneous liver biopsy group. It is important to note that some studies included in this meta-analysis used FNA or earlier generation biopsy needles, which are no longer felt to be ideal for obtaining optimal tissue. A similar meta-analysis comparing EUS-LBx to both percutaneous and transjugular approaches included 5 studies with over 500 patients and concluded that, based on the subgroup analysis limited to EUS biopsy needles currently in clinical practice, there was no difference in biopsy adequacy or adverse events for EUS-LBx compared to percutaneous and transjugular routes.^13^

**FIGURE 2:**
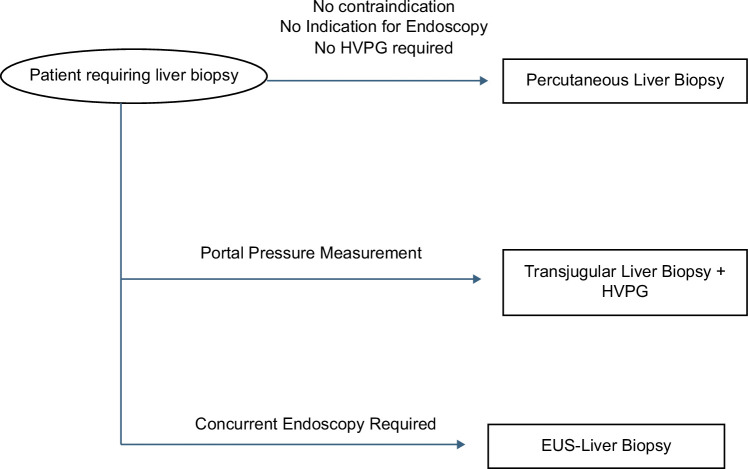
Proposed clinical decision pathway/algorithm for EUS-guided liver biopsy. Abbreviation: EUS, endoscopic ultrasound.

While EUS-LBx is increasingly used, it remains unclear if this should be the first-line approach to sampling hepatic parenchyma in patients. IR-guided biopsy often involves minimal sedation, can be coupled with HVPG gradient and may be associated with lower costs. For those reasons, we suggest the following flowchart for when to consider EUS-LBx to ensure appropriate resource utilization and patient satisfaction (Figure 2).

### EUS-guided focal liver lesion biopsy

**FIGURE 3:**
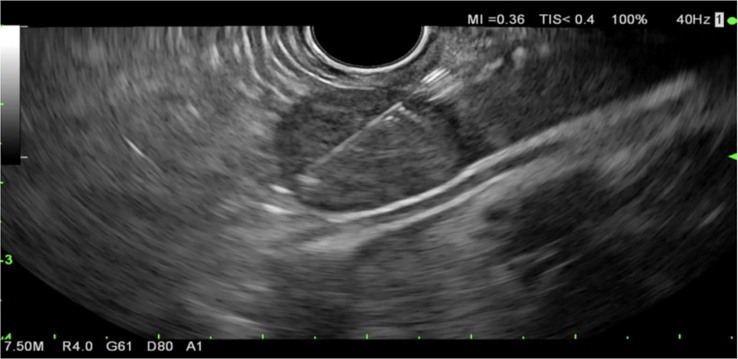
EUS-guided core biopsy of an isoechoic lesion in the left lobe of the liver. Abbreviation: EUS, endoscopic ultrasound.

Echoendoscopy has also been increasingly used for targeted biopsies of hepatic lesions, as linear echoendoscopes allow for safe needle access for sampling.^14^,^15^ The liver is usually scanned from the stomach to the duodenum, and most segments of the liver can be visualized endosonographically. The left lobe and its segments can be evaluated from a transesophageal/transgastric view, while the right lobe is usually assessed from a transduodenal approach, including segments V, VI, VII, and VIII in addition to segment IV of the left lobe.^14^,^15^,^16^ In a similar fashion to fine needle biopsy of pancreatic masses or abdominal lymphadenopathy, the needle is passed into the target lesions under suction or via the stylet pull method (Figure 3). It is imperative that a multidisciplinary discussion has been performed with hepatology, interventional radiology (IR) and surgical oncology to ensure the most appropriate method of sampling is employed. Historically, lesions in the left lobe of the liver can be difficult for IR access, and more suitable for EUS-guided access.

EUS has shown particularly promising results in the diagnosis of primary HCC and metastatic disease to the liver. In one small study evaluating HCC and metastatic disease, EUS was able to detect hepatic lesions ranging in size from 0.3 cm to 14 cm, with EUS identifying new or additional lesions in 28% of the patients, all <0.5 cm in size. Additionally, EUS-FNA was performed in more than 70% of the patients, all of whom yielded a clinical diagnosis.^17^ In a Veterans Affairs (VA) study from Texas, 132 patients with newly diagnosed pancreatic, biliary, esophageal, colon, gastric, or lung cancer were prospectively enrolled in a study comparing EUS to CT scans for the detection of liver metastases. CT scan detected metastases in 26/132 patients, while EUS detected liver lesions in 35/132, of which 32 underwent fine needle aspiration/biopsy and 24 had confirmed diagnosis of metastases. The diagnostic accuracy of EUS/EUS-FNA and CT scan was therefore 98% and 92%, respectively (*p*=0.0578).^18^

### EUS-guided coil injection therapy for gastric varices

#### Introduction

Gastric varices (GV) account for 10%–20% of all variceal bleeding, but can be associated with significantly higher morbidity, severity of bleeding, and higher risk of rebleeding.^19^ The prevalence of GV ranges between 17% and 25% among patients with cirrhosis^20^ GV most commonly occurs from portal hypertension and vascular shunt formation. However, GV can also be seen in non-shunt dependent pathology, such as splenic vein thrombosis, commonly seen in pancreatitis or pancreatic cancer.^21^ While the management of GV involves a multidisciplinary approach with various invasive and noninvasive medical interventions, endoscopy has played an increasing role in the diagnosis, and more recently, treatment of GV.

Endoscopic management of GV has been premised on the direct endoscopic injection of glue and acrylate polymers to induce thrombosis, cessation of bleeding and reduction in risk of rebleeding. While this technique is successful in bleeding cessation and remains a recommendation by societal guidelines,^20^ it carries a risk of rebleeding (immediate and delayed), can be damaging to endoscopes and can result systemic embolization which can be seen in 1%–2% of cases (brain, lung, spleen, and others).^22^,^23^,^24^,^25^,^26^,^27^ Additionally, cyanoacrylate glue may not be readily available at all institutions, limiting the widespread use of this endoscopic modality.

Echoendoscopy has revolutionized the management of GV by providing a new tool in the armamentarium for patients with GV. EUS allows for real-time assessment or “varicealography” of GV by evaluating size, flow and presence of a feeder vessel. EUS also allows for therapeutic intervention through EUS-guided glue and/or coil injection therapy with simultaneous assessment of response to therapy in real time via Doppler flow.

#### Literature/supporting data

Studies have shown that EUS-guided cyanoacrylate injection is superior to direct injection therapy for bleeding GV.^28^ In this study by Bick et al comparing EUS-CYA injection (64 patients) to direct endoscopic CYA injection (40 patients), EUS-CYA injection used less cyanoacrylate per procedure but targeted more varices than direct injection therapy. Patients in the EUS group had lower rates of gastric variceal rebleeding at 30 days (8.8% vs. 23.7%); however, no differences were seen in subgroup analysis in patients with cirrhosis. Adverse event rates were similar between both groups, while mortality was not assessed.

EUS cyanoacrylate injection, however, carries the same risk associated with glue injection. To mitigate these risks, EUS-guided coil injection therapy has emerged as a safe and exciting option for the management of GV. In 2008, Levy et al^29^ were the first to apply the use of EUS-guided coil embolization of GV. Since then, this technique has been increasingly popular with mounting literature supporting its use. This technique entails the injection and packing of GV with hemostatic coils to allow for thrombosis and obliteration of vascular flow. The coils are thought to act as a scaffold to assist with glue or other injectable therapy, thereby reducing the theoretical risk of embolization. This approach has been associated with high clinical and technical success rates and low rates of adverse events, particularly when compared to cyanoacrylate injection therapy.^29^,^30^,^31^,^32^,^33^,^34^

EUS-guided coil therapy has arguably emerged as the new face of endohepatology. This technique has been shown to be efficacious as prophylactic or initial therapy for bleeding GV or when alternative therapies such as IR-guided interventions are limited. Recent large multicenter studies have shown promising real-world data for EUS-GV coil therapy. Bazarbashi et al demonstrated a technical success rate of 100%, a clinical success rate (cessation of bleeding at 1 month) of 88%, and a low adverse event profile in the first US multicenter experience on EUS-guided coil therapy. That same year, in 2023,^35^ Samanta et al published a multicenter, comparative, international study including 276 patients who underwent EUS-guided coil therapy versus endoscopic cyanoacrylate injection for GV active bleeding, stigmata of recent bleeding from GV and primary prophylaxis. This study revealed that EUS-coil therapy was associated with significantly fewer number of required sessions, subsequent bleeding episodes and re-intervention rates when compared to endoscopic cyanoacrylate injection.^36^ Active bleeding was seen in 7% of the EUS-coil group and in 21% of the direct endoscopic therapy group, while stigmata of recent bleeding were present in 62% of the EUS-coil group and 50% of the direct endoscopic therapy group. More recently, a meta-analysis evaluating EUS-guided therapies for GV, which included over 600 patients from 18 studies, confirmed that EUS therapies are highly effective, technically feasible and clinically safe for managing primary prophylaxis or secondary prophylaxis of GV.^37^ In this meta-analysis, the overall pooled rate of gastric variceal obliteration (on upper endoscopy and EUS) was 90.2% and the pooled rate of post-therapy bleeding of 4.9% (all after 30 days). For secondary prophylaxis, the pooled rate for GV obliteration was 83.6%, with pooled rates of GV rebleeding and recurrence of 18.1% and 20.6%, respectively.

#### Technique

**FIGURE 4:**
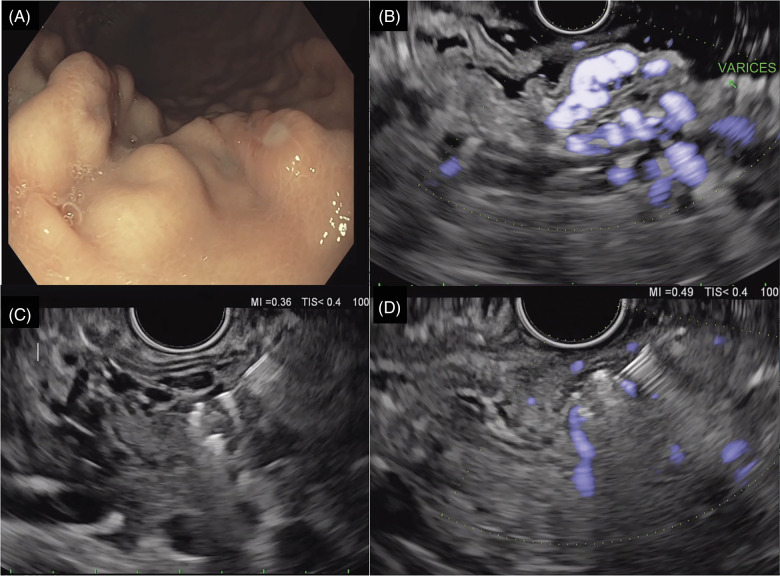
(A) Gastric varices with evidence of white nipple sign indicating recent bleed. (B) EUS-guided Doppler evaluation of gastric varices. (C) EUS-guided deployment of coils into gastric varices. (D) Significant reduction in Doppler flow after coil deployment into gastric varices. Abbreviation: EUS, endoscopic ultrasound.

During EUS-guided coil therapy, patients are placed in the left lateral decubitus position under general anesthesia and given a dose of prophylactic antibiotics. An endoscopy is performed to evaluate the GV and confirm the accurate subtype (IGV-1 or GOV2). It is recommended that 200–250 mL of saline or water be instilled into the gastric fundus to improve visualization of submucosal varices and distinguish them from extra-gastric collaterals that may be present. GVs are measured under EUS guidance, and the Doppler flow is studied. Coil diameter and length are selected, with 0.018″ and 0.035″ coils passing through 22 G and 19 G fine aspiration needles, respectively. It is recommended that the length of coils should be at least 30%–50% of the diameter of the variceal nests being targeted. Coils are placed while the needle is in the target varices by advancing a stylet, and repeated until additional coils cannot be advanced or a significant reduction in Doppler flow is seen (Figure 4). It is imperative that the operator and assistant practice coil reloading to ensure a smooth process throughout the procedure. Contrast can be injected to ensure no evidence of “run-off” through a large shunt, which may increase the risk of embolization. After coils are placed, the operator determines whether to perform concurrent injection, such as glue or absorbable gelatin sponge. Fluoroscopy can assist with this intervention as coils can be seen filling the lumen of the variceal nests. While there is variability in clinical practice, it is typically recommended to repeat an EUS at 1, 3, and 6-month intervals, with additional coils placed if residual flow exists in GV.^38^,^39^,^40^

While systemic embolization of glue has been well documented and occurs at a rate of 1%–2% in published literature, there is sparse data on coil migration and embolization and predominantly exists in the form of case reports.^41^,^42^ Alternatives to glue or acrylate polymers that can be injected after coil therapy include thrombin, absorbable gelatin sponge (Surgiflo or Gelfoam), and RADA16 self-assembling peptide (Purastat 3D Matric). Data comparing these different modalities is lacking.^43^,^44^,^45^

**FIGURE 5:**
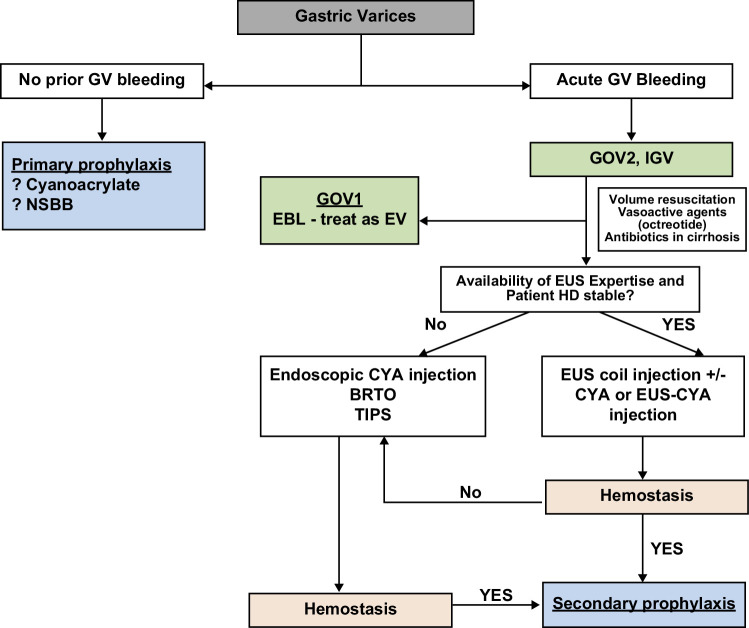
Proposed clinical decision pathway/algorithm for EUS-guided coil injection therapy for gastric varices. Abbreviation: EUS, endoscopic ultrasound.

While EUS-guided coil therapy has emerged as a promising therapy for GV, many unanswered questions remain regarding the injectates of choice to use in conjunction with coil therapy, the relative contribution of coils to glue or other injectates, the ideal number and length of coils, and whether to target the varices or the perforator feeder vessels. Numerous studies to answer these questions are underway. Here, we propose an algorithm that may assist in deciding when to proceed with EUS-guided coil therapy for gastric variceal bleeding (Figure 5).

### Endoscopic management of refractory esophageal variceal bleeding

Endoscopic management of esophageal variceal bleeding has been premised on endoscopic band ligation, which is safe and effective for the treatment of variceal bleeding.^20^ However, in some instances, endoscopic band ligation may not be sufficient in controlling bleeding or limited in its ability to control bleeding. Recommendations have evolved over time to manage refractory acute esophageal variceal bleeding with the goal of providing temporary hemostasis as a bridge to definitive portal pressure-reducing therapy, such as TIPS placement.^20^

Historically, and to date, balloon tamponade therapy such as Sengstaken-Blakemore or Minnesota tubes is recommended for managing refractory esophageal variceal bleeding, with immediate hemostasis rates of over 90%.^46^,^47^ While these esophageal tamponade tubes are effective, they are associated with several limitations. Risks of tube inflation include esophageal necrosis (if left in place for longer than 24–48 h), ulceration and aspiration. It is imperative that training in esophageal balloon dilation is performed, as complications such as perforation can occur with incorrect tube placement or inflation.^48^

**FIGURE 6:**
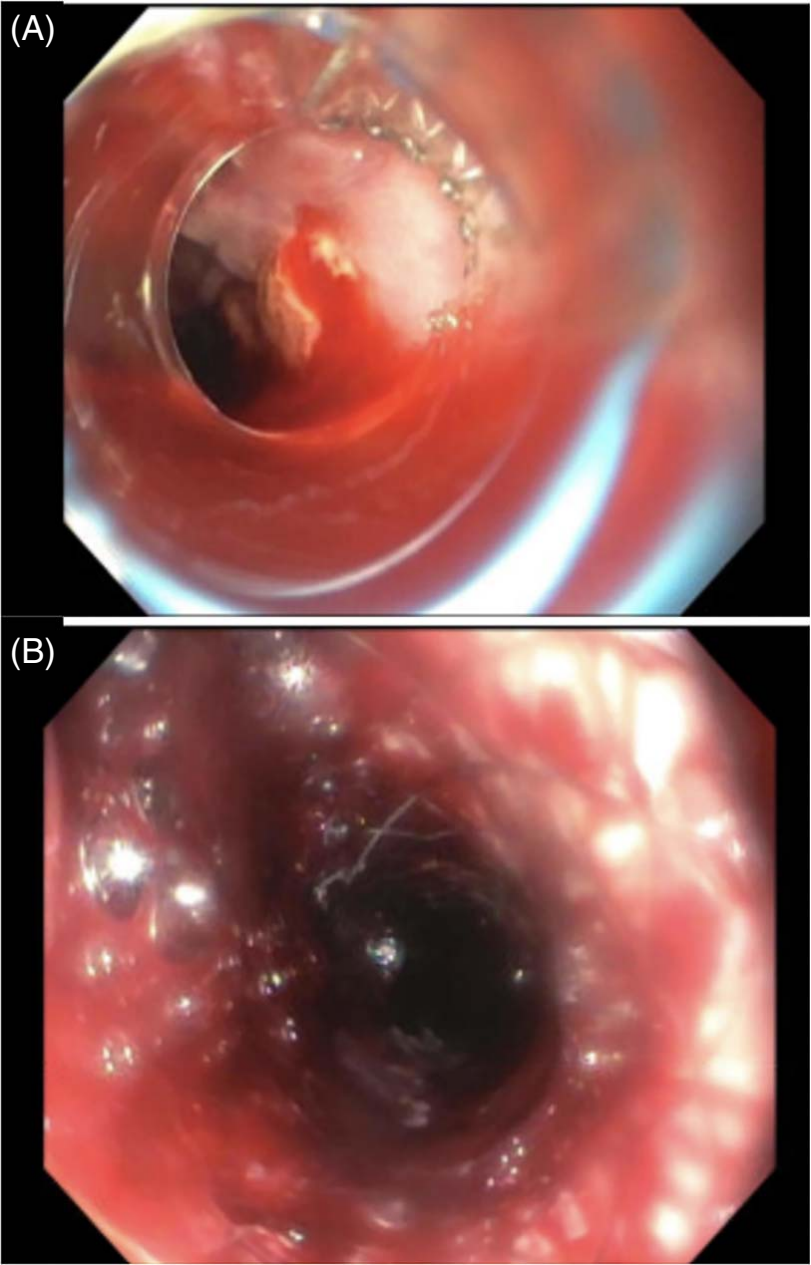
Stent placement for management of refractory esophageal variceal bleeding after failed band therapy. Refractory esophageal variceal bleeding despite band ligation attempt (A) which is subsequently treated with endoscopic SEMS placement (B).

Esophageal self-expanding metal stent (SEMS) therapy has emerged as an alternative, and perhaps a better option, where expertise and resources are available. Esophageal stent therapy for tamponade of refractory variceal bleeding is an off-label use in the United States. These stents, commonly used for malignant esophageal obstruction, provide a tamponade effect through their self-expanding nature and thereby provide a bridge to TIPS (Figure 6). A randomized controlled trial by Escorsell et al^49^ found that esophageal SEMS provided greater hemostasis when compared to balloon tamponade therapy (66% vs. 20%) with fewer serious adverse events (15% vs. 47%). Common adverse events of stent placement include stent migration, which can be seen in up to 38% of cases.^50^

It is important to note that some stents, available in Europe, have been designed and specifically cleared for variceal bleeding. These are not available for use in the United States. It is also important to note that AASLD guidelines recommend either balloon tamponade therapy or stent placement for refractory acute variceal bleeding,^20^ while BAVENO working group comments that SEMS are as efficacious but safer than balloon tamponade.^51^

While EV bleeding-specific esophageal stents do not exist in the United States yet, off-label use of esophageal stents for malignancy can be used in patients with refractory EV bleeding. Through-the-scope esophageal metal stents (such as Boston Scientific Agile or Taewoong Niti-S) are quite easy to deploy and can be advanced through the working channel of a 1T endoscope; they lack significant radial force when compared to over-the-wire esophageal stents such as Boston Scientific Wallflex esophageal stents or Olympus Hanarostents. While fluoroscopy assists with stent localization, this is not required, particularly when these need to be deployed in a timely fashion. Additionally, while stent fixation will assist with migration, this is not necessary, as most of these stents will be removed within few days of placement once hemorrhage has been definitively controlled.

### The role of EUS and ERCP in patients undergoing liver transplantation

Endoscopic intervention plays an important role in the management of patients prior to and following liver transplantation.^52^ Prior to transplant, endoscopic interventions include predominantly screening/surveillance and/or treatment of esophageal or GV, in addition to endoscopic retrograde cholangiopancreatography (ERCP) for the management of biliary strictures. Following transplant, endoscopic care focuses mainly on the evaluation of abnormal liver tests, particularly those related to biliary complications.

**FIGURE 7:**
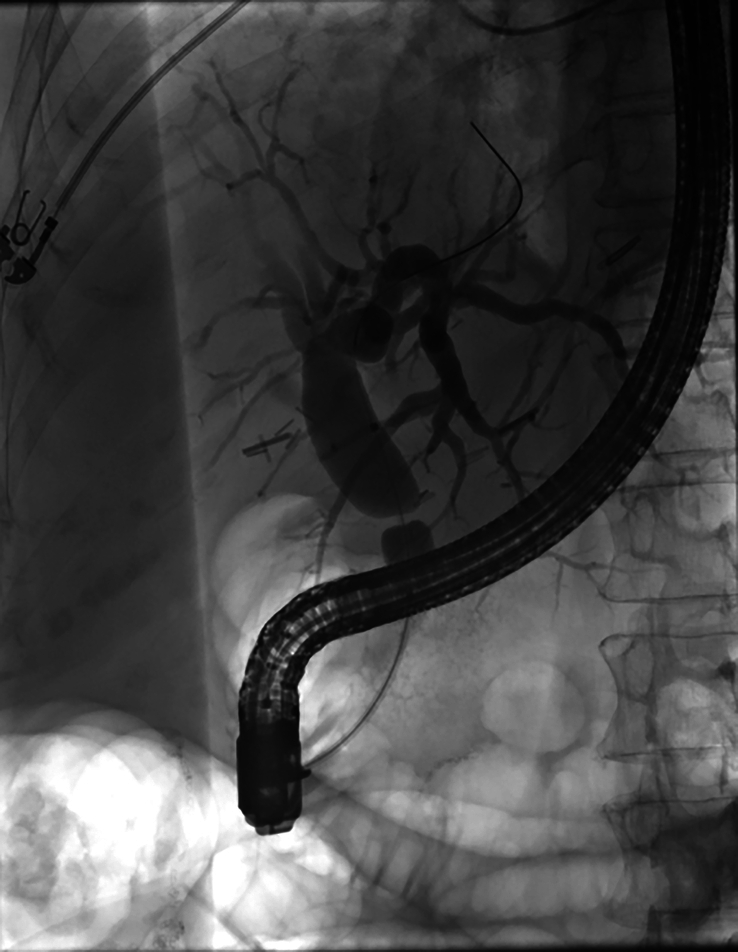
ERCP revealed a severe anastomotic stricture at the bile duct in a patient post-liver transplantation. Abbreviation: endoscopic retrograde cholangiopancreatography.

Biliary pathology is seen frequently in patients following liver transplant, the most common of which are bile leaks and biliary strictures. Biliary strictures range from focal anastomotic strictures to diffuse biliary strictures from ischemia or thrombosis^53^ (Figure 7). These strictures can lead to significant morbidity and oftentimes require endoscopic interventions.

ERCP is often employed in both bile leaks and strictures and has been proven to be highly efficacious in treating these complications, with success rates ranging from 75% to 100% (higher success rates with bile leak management vs. strictures).^54^,^55^,^56^,^57^ While standard of care management for anastomotic bile duct strictures has been premised on dilation (depending on timing since transplant) and step-wise plastic stent placements, recent recommendations from the American Society of Gastrointestinal Endoscopy (ASGE) suggest several advantages to the use of covered metal stents for anastomotic strictures.^58^ However, it is acknowledged that several factors influence the type and number of stents used during ERCP including the time since liver transplant, stricture distance from both the bifurcation and the ampulla, length of common bile duct, concern for jailing off a branch of the bile duct, stent availability, risk of pancreatitis with metal stents, local expertise, and cost.

EUS also plays a role in the management of patients following liver transplant. Similar to EUS-LBx of native liver parenchyma, fine needle biopsy can be safely performed in the transplanted patient.^59^ Special considerations include reviewing cross-sectional imaging to ensure safe access to the transplanted liver (via transgastric or transduodenal view) and administration of antibiotic prophylaxis given immunosuppression.

EUS can also be coupled with same-session ERCP for the evaluation of abnormal liver function tests to streamline patient care. For example, if an ERCP reveals no stricture or improvement of a stricture after stent placement; however, abnormal liver function tests are still present, EUS can be added at the same session for parenchymal liver biopsy. Han et al^60^ recently described the safety and feasibility of same-session concomitant EUS-LBx and ERCP. In a single-center retrospective study examining post-liver transplant patients with abnormal liver function tests, these authors discovered that 50% of patients had both rejection and anastomotic stricture.

### Additional roles for EUS in patients with liver disease

There are several emerging roles for EUS in patients with liver disease. EUS can be very sensitive in detecting ascites, which is seen as anechoic compartments of fluid surrounding the liver or below the diaphragm. EUS can be used to perform FNA to assess ascitic fluid for cell count, cytology, protein and/or microbial analysis. A study by Nguyen et al confirmed that EUS was sensitive for the detection of ascites, and in some cases more sensitive than cross-sectional imaging, and also safe for fluid aspiration.^61^,^62^ While not standard of care, this option may offer an alternative for patients when standard methods of ascites detection and aspiration are not feasible or limited, and particularly when malignant ascites is suspected.^61^,^62^

EUS also has an emerging role in the management of liver abscesses.^63^,^64^ While percutaneous drainage is predominantly performed with interventional radiology, some abscesses, particularly those in the left lobe, may be difficult to reach. EUS can provide an alternative route for sampling and transmural drainage. While not feasible for right-sided abscess, EUS-guided liver abscess drainage has been associated with quicker resolution of symptoms, shorter length of hospital stay, fewer adverse events, and requires fewer procedural sessions when compared with the percutaneous technique.^65^

## FUTURE AND NOVEL DIRECTIONS IN ENDOHEPATOLOGY

Endohepatology has been gaining momentum with the evolution of endoscopic technology. While many of these approaches are being used in practice, the majority of these interventions are experimental and require more research to confirm safety, feasibility, and clinical meaningfulness prior to adopting them into guideline management and routine clinical practice.

### EUS-guided hepatic elastography

**FIGURE 8:**
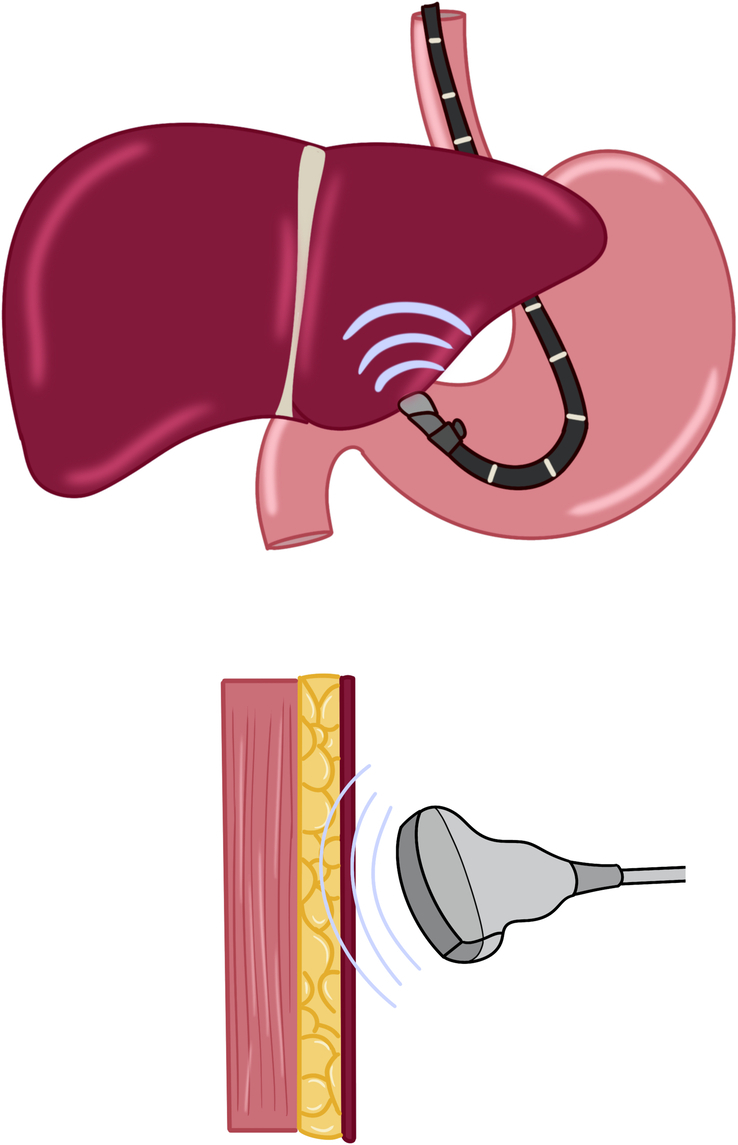
Schematic representation of EUS-guided liver elastography. Abbreviation: EUS, endoscopic ultrasound.

**FIGURE 9:**
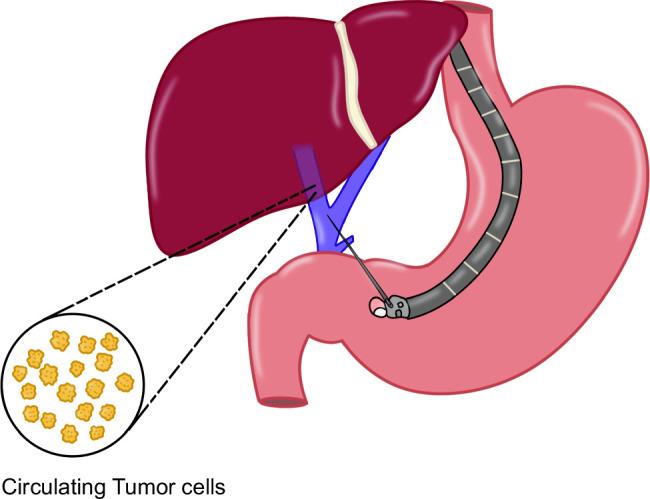
Calculation of liver stiffness using EUS-guided elastography. Abbreviation: EUS, endoscopic ultrasound.

Transabdominal transient elastography (FibroScan) has revolutionized noninvasive liver disease assessment by providing a “virtual biopsy” to evaluate hepatic steatosis and fibrosis.^66^ It measures liver stiffness using shear wave velocity, but its accuracy can be affected by factors such as obesity, ascites, and hepatic congestion.^66^ EUS-guided shear wave elastography (EUS-SWE) has emerged as a promising alternative by bypassing abdominal adiposity, potentially improving liver stiffness measurements in patients with high BMI (Figures 8, 9). Additionally, liver stiffness measurement and transient elastography can be associated with significant intraobserver and interobserver variability.^67^ While not routine in common clinical practice, some data regarding EUS-elastography have been reported. Studies have demonstrated a strong correlation between EUS-SWE and fibrosis, with comparable accuracy to transient elastography. Additionally, EUS-SWE has shown superiority over the fibrosis-4 index (FIB-4) score and transient elastography in detecting advanced fibrosis and cirrhosis.^68^,^69^,^70^ In a recent multicenter cross-sectional study, Wang et al evaluated EUS-SWE compared to FIB-4 score and transient elastography with liver biopsy as gold standard. EUS-SWE was superior to FIB-4 in discriminating significant fibrosis and advanced fibrosis but not cirrhosis while EUS-SWE was superior to transient elastography in predicting advanced fibrosis and cirrhosis.^70^ While promising, further research is needed to standardize data acquisition techniques and define its clinical applications.

### EUS-guided portal venous sampling

**FIGURE 10:**
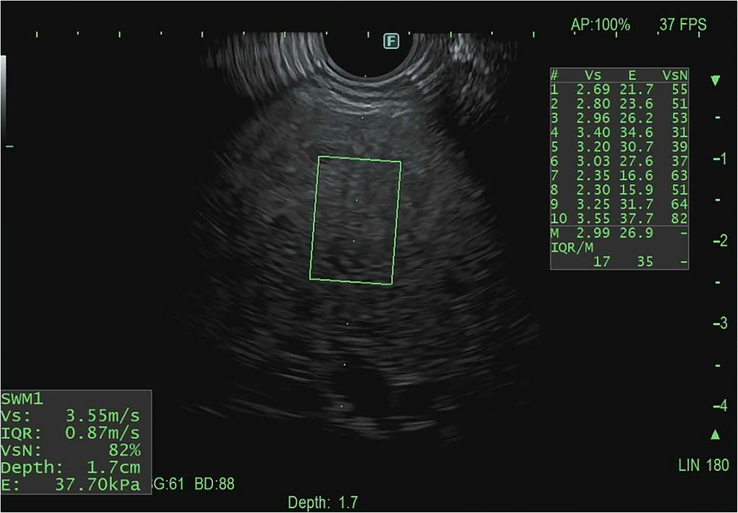
Schematic representation of EUS-guided portal venous sampling. Abbreviation: EUS, endoscopic ultrasound.

The portal vein directs blood from the pancreas, spleen, and gastrointestinal (GI) tract to the liver, carrying nutrients, microbiota, and metabolic products.^71^ In GI malignancies, circulating tumor cells (CTCs) in the portal vein present an opportunity for liquid biopsy analysis. While percutaneous and surgical portal vein access can be invasive, echoendoscopy (EUS) has emerged as a less invasive alternative and has emerged as an intervention for sampling of CTCs^72^ (Figure 10). First described in 2015, EUS-guided portal venous sampling demonstrated 100% CTC detection in patients with pancreaticobiliary malignancies, outperforming peripheral blood sampling.^73^ Subsequent studies confirmed its feasibility, prognostic value, and technical success.^74^,^75^ Beyond oncology, EUS-guided portal venous sampling may offer insights into metabolomics, microbiome analysis, and liver disease. Further research is needed to standardize techniques and expand their clinical applications.

### ERCP and EUS-guided injection therapies

EUS-guided chemoembolization and endoscopic liver-directed gene therapy represent experimental treatments for patients with liver tumors. These treatments require that the liver lesions are accessible to EUS, and therefore may have a particular impact on tumors in the left lobe of the liver. Experimental real-world EUS-guided therapies for hepatic tumors, including metastasis, include the injection of ethanol, YAG laser ablation and radiofrequency ablation.^76^,^77^,^78^,^79^,^80^

**FIGURE 11:**
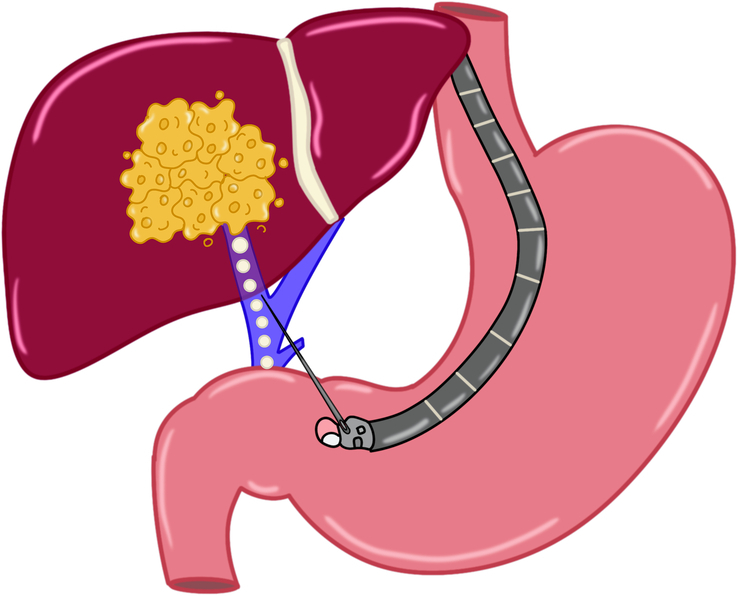
Schematic representation of EUS-guided portal chemoembolization. Abbreviation: EUS, endoscopic ultrasound.

In more recent years, experimental animal studies have revealed promising treatment through EUS guidance. In a preclinical proof of concept in a study in pigs, Faigel et al demonstrated EUS-guided delivery of chemotherapy-eluting microbeads into the portal vein (Figure 11). Technical success was 100%. EUS-guided portal vein injection of irinotecan resulted in twice the hepatic concentration and half the systemic concentration. Additionally, liver histology confirmed the presence of microbeads within small portal venules.^81^ In another endohepatology-based investigational study, Khumbari et al demonstrated an ERCP-based biliary approach for the delivery of DNA plasmids into the bile duct in a live pig model. This was associated with a 100% technical success rate without adverse events. Genomic integration and protein expression were observed in targeted liver tissue in animal models surviving to 60 days.^82^ Finally, EUS-guided portal vein embolization has been described to allow compensatory hypertrophy of the contralateral lobe prior to resection for hepatic malignancy. This technique has been performed in experimental pig models using either EUS-guided microcoils or ethylene-vinyl alcohol copolymers.^83^,^84^

### EUS-guided portal pressure gradient measurement

**FIGURE 12:**
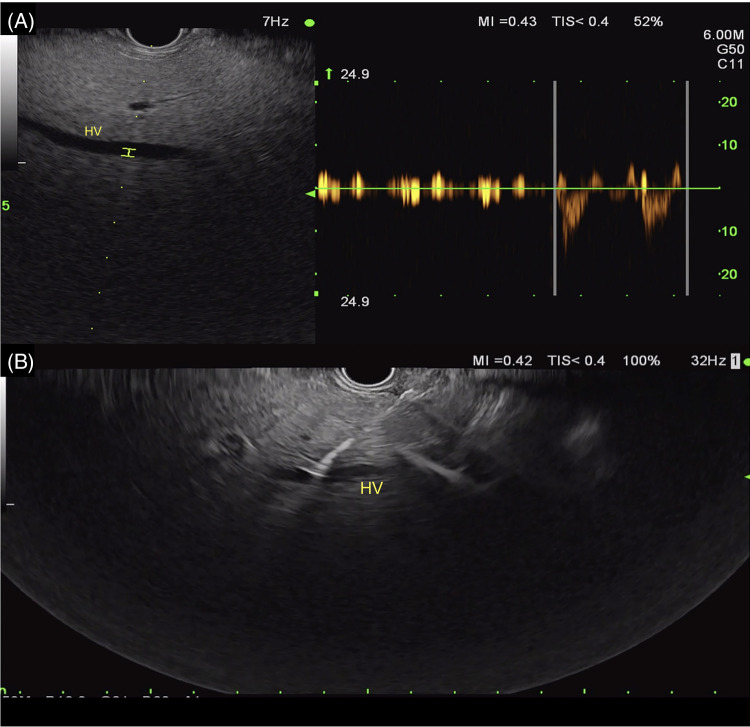
EUS-guided portal pressure measurement of the middle hepatic vein. Abbreviation: EUS, endoscopic ultrasound.

**FIGURE 13:**
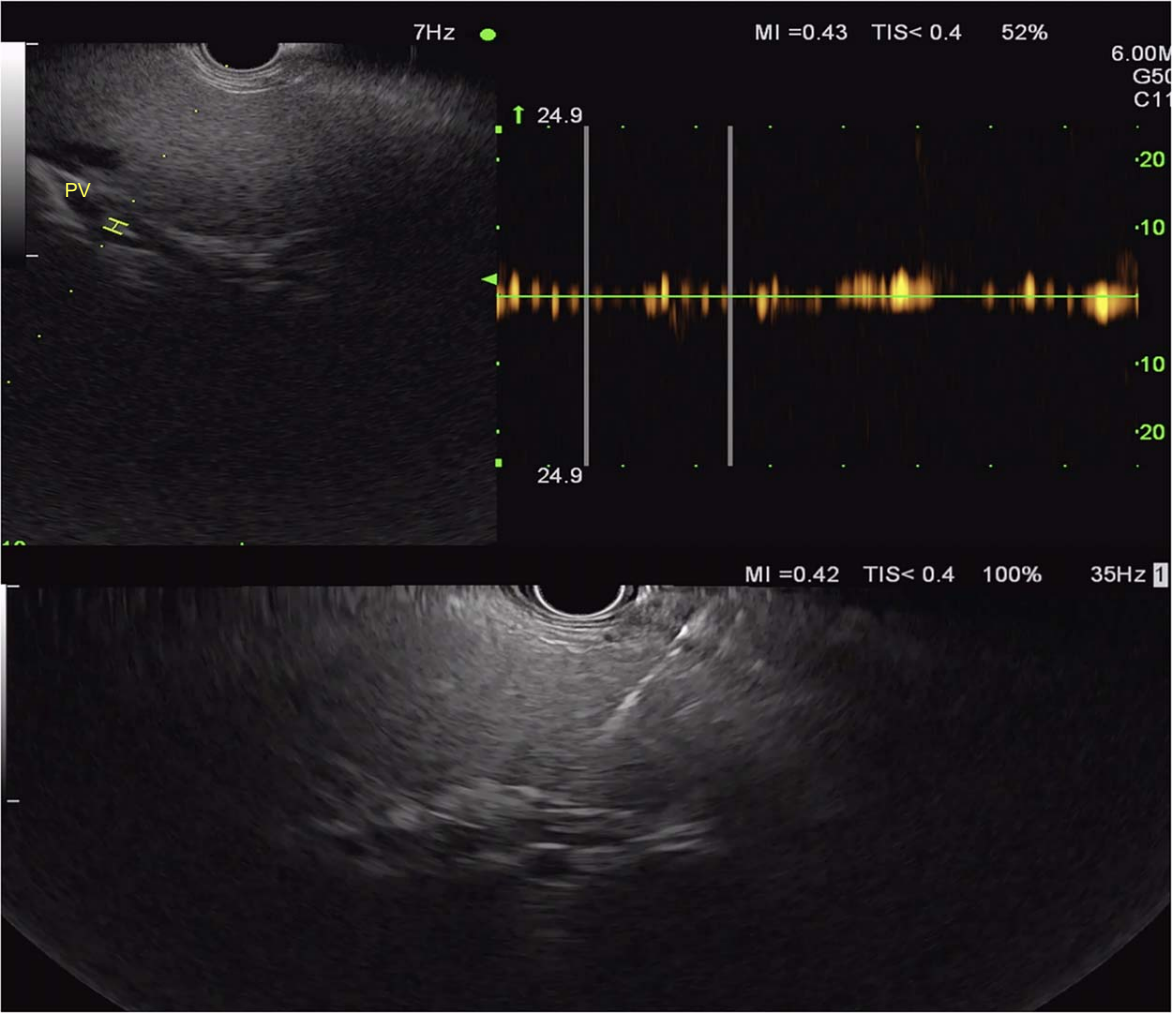
EUS-guided portal pressure measurement of the portal vein. Abbreviation: EUS, endoscopic ultrasound.

**FIGURE 14:**
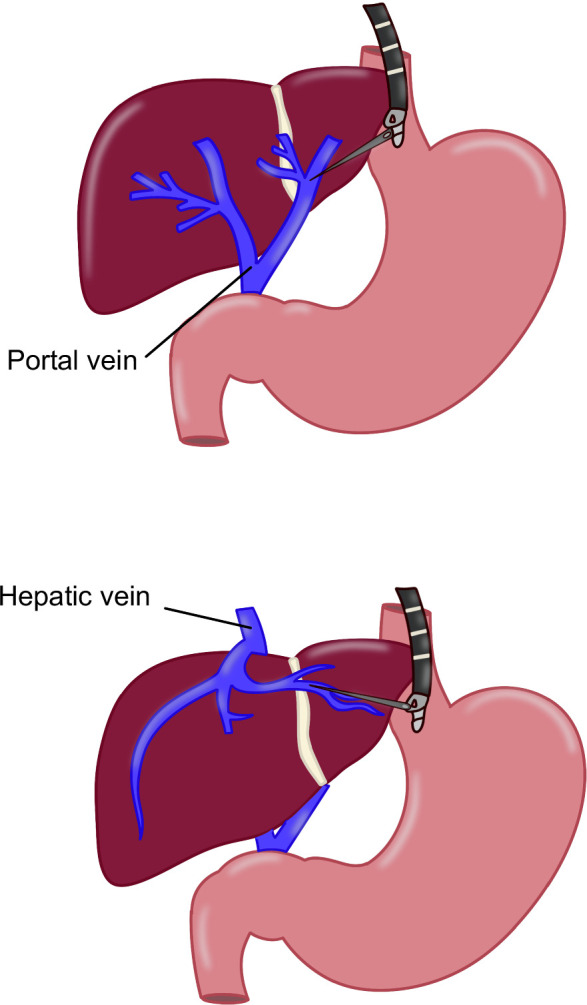
Schematic representation of EUS-guided portal pressure gradient measurement explaining needle access into each vessel. Abbreviation: EUS, endoscopic ultrasound.

**FIGURE 15:**
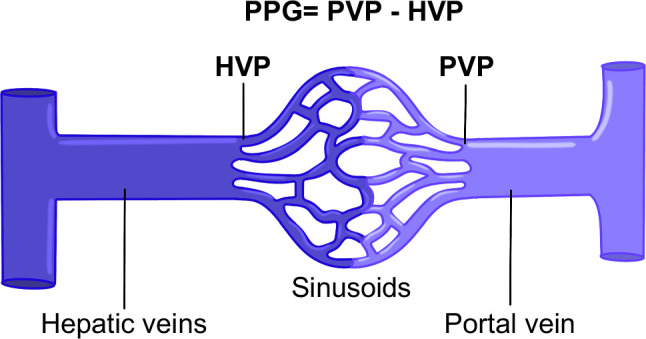
Portal pressure gradient measurement. Abbreviations: HVP, hepatic vein pressure; PPG, portal pressure gradient; PVP, portal vein pressure.

EUS-guided portal pressure gradient measurement (EUS-PPG), first introduced in animal models and later in humans in 2016, estimates portal hypertension by measuring the gradient between the portal and hepatic systems via EUS-guided needle manometry.^85^,^86^,^87^ The procedure involves advancing a 25 G needle transgastrically into the middle hepatic vein and then the intrahepatic portal vein to calculate the pressure difference (Figures 12–15). While still experimental, studies have demonstrated its feasibility, safety, and correlation with clinical cirrhosis and portal hypertension parameters.^88^,^89^ A meta-analysis of 8 studies (178 patients) reported a 94.6% technical success rate and 85.4% clinical success rate with minimal adverse events.^89^ The only comparative study to date compared EUS-PPG to the gold standard HVPG and revealed promising results. Despite including only 33 patients, the correlation between the 2 techniques showed an intraclass correlation coefficient of 0.82 (0.65–0.91) with major discrepancies of >5 mm Hg seen in only 4 patients. No differences in adverse events were observed.^90^ Many questions still remain unanswered, such as the reproducibility of this technique and the impact of sedation (need for EUS) on pressure results. Though further research is needed, EUS-PPG may serve as a valuable tool, particularly for patients already undergoing upper endoscopy or liver biopsy, to better assess portal and assist in guiding clinical care when portal hypertension may remain equivocal.

### EUS-guided portosystemic shunt therapy

EUS-guided portosystemic shunt therapy, an experimental procedure studied exclusively in animal models, involves creating a vascular connection between the portal and hepatic veins using a lumen-apposing metal stent (LAMS) under EUS guidance, mimicking IR-guided shunt creations or TIPS. Animal studies have demonstrated technical feasibility but highlighted challenges such as stent thrombosis and mispositioning, necessitating further advancements in delivery systems and better assessment of possible clinical applications.^91^,^92^,^93^,^94^

### Endobariatrics and liver disease

Endohepatology has evolved to encompass the field of endobariatrics given the overlap of obesity and liver disease, specifically metabolic dysfunction–associated steatotic liver disease (MASLD). Endobariatrics includes primary weight loss procedures such as endoscopic sleeve gastroplasty (ESG) and other gastric remodeling procedures, revisional endoscopic weight loss therapies such as transoral outlet reduction (TORe) or sleeve-revisions in patients following bariatric surgery, and investigational small bowel interventions aimed to treat the comorbidities of obesity such as small bowel ablation, duodenal liners, or diversion techniques.

The MERIT trial, a recent multicenter RCT evaluating endoscopic sleeve gastroplasty (n=85) versus lifestyle modification (n=124) in patients with classes I and II obesity found the mean percentage of excess weight loss in patients assigned to ESG versus the control group was 49·2% and 3.2%, respectively (*p*<0.0001). The percent total body weight loss was 13.6% and 0.8% for the ESG versus control group, respectively (*p*<0.0001).^95^ Patients who underwent ESG also had significant improvement in liver transaminases, hepatic steatosis index, and aspartate transaminase-to-platelets ratio index.

Furthermore, in one meta-analysis of 18 studies with over 850 patients undergoing endoscopic bariatric and metabolic therapies, mean weight loss was 14.5% at 6 months. Additionally, liver fibrosis was significantly reduced by a standardized mean difference (SMD) of 0.7. Finally, there was improvement in NAFLD surrogates, including ALT, hepatic steatosis, and histologic NAFLD activity score.^96^

Jirapinyo et al^97^ revealed that combination therapy, with endoscopic gastric remodeling (EGR) and GLP-1 agonists yielded greater improvements in fibrosis compared to EGR monotherapy group [ALT: reduction by 55±23% vs. 29±22% (*p*=0.008), NAFLD fibrosis score: reduction by 181±182% vs. 30±83% (*p*=0.04), liver stiffness measurement on transient elastography: reduction by 54±12% vs. 14±45% (*p*=0.05)] in addition to a larger reduction in weight loss [18.2±6.6% total weight loss (TWL) vs. 9.6±3.3% TWL (*p*=0.004)].

Endoscopic bariatric and metabolic therapy has shown improvement in MASLD parameters and reduction in fibrosis. One prospective study including 45 patients with obesity and MASLD with clinically significant hepatic fibrosis (fibrosis stage ≥F2) demonstrated improvement in multiple noninvasive tests of hepatic fibrosis.^51^ Reductions in ALT (49.7 U/L down to 24.2 U/L), AST (39.1 U/L down to 24.1 U/L), NFS (0.5±1.5 to −1.2±1.6), FIB-4 index (1.4 down to 1.2), vibration-controlled transient elastography (13.9 kPa down to 8.9 kPa), and Agile 3+ fibrosis score (0.5 down to 0.4) were found 6–12 months after the procedure^98^ Furthermore, one small study including 20 patients with MASLD and compensated advanced chronic liver disease indicated improvement in portal pressure measurement after gastric remodeling procedures. Patients enrolled underwent EUS-guided PPG prior to and 6 months following endoscopic gastric remodeling using a plication device for weight loss. At 6 months, median PPG decreased from 5.4 mm Hg (range 0.7–19.6) to 1.8 mm Hg (range 0.4–17.6) (*p*=0.002), with 79% of patients experiencing ≥20% reduction in PPG. There was also a significant improvement in noninvasive tests of liver fibrosis.^99^

## CONCLUSIONS

Endohepatology is an exciting and expanding field with a rapidly changing landscape. The growing endoscopic armamentarium has led to numerous applications for endoscopy and echoendoscopy in the diagnosis and management of patients with liver disease, oftentimes allowing for simultaneous or concurrent interventions to facilitate streamlined testing, appropriate resource allocation, and improved patient satisfaction. While several of these interventions have demonstrated clinical impact and are being performed routinely at centers with expertise, many remain investigational or experimental, with more research required to confirm clinical utility and/or clinical equivalence to existing approaches. As the field of endoscopy continues to evolve, endohepatology will undoubtedly play an increasingly important role in the management of patients with liver disease.
